# Case of Polypus of the Vulva

**Published:** 1839-03-16

**Authors:** David M. Wright

**Affiliations:** Edenton, N. C.


### CASE OF POLYPUS OF THE VULVA.

By David M. Wright, M. D.

*January 17th.*—I was called a few miles in the country to visit a negro girl, about 16 years of age, who was represented to be in a very perilous condition. On my arrival, I was told by the mother of the girl, that her womb had fallen from her, and was then hanging by a chord several inches from the os externus. On examination, what had been mistaken for the womb was ascertained to be a polypus. The chord or pedicle by which it was suspended, was about two inches in length, half an inch in diameter, and round, except at the base or root, where it was flat, and probably an inch in width. The tumour was spherical, and resembled very much an Irish potato before the pealing is removed, having places corresponding in appearance with what are called eyes in the potato, and produced no doubt by the rupture and drying up of some small vesicles on its surface, some of which still remained unbroken. On removing the tumour, which was done by a few cuts with the scalpel, but slight hæmorrhage occurred, not sufficient to render a ligature necessary, nor was the operation productive of much pain. The tumour after removal was as large as an orange, and weighed seven ounces. It was quite translucent, and being cut into, presented a cellular appearance, the cells being filled with a glairy fluid like white of egg. I learned on inquiry that the tumour had fallen from the vagina about ten days previously, while she was at work in the field, and that she had continued to go about as usual for several days with it suspended by the pedicle; the parts at length becoming exquisitely tender and painful, she was compelled to resume the horizontal position, and reveal her situation. Her general health had been very good, and the only inconvenience experienced was a slight pain when her legs were pressed tightly together; and this had been observed only about seven or eight weeks, so that the tumour had probably not been much longer in attaining its growth.

The most remarkable peculiarity in the tumour was its place of attachment, being at the upper portion of the labia interna. I do not remember to have seen a description of one having similar attachment, and it is difficult to imagine, having its attachments as described, the manner in which it could have gained its position in the vagina, or retained it, having gotten there.

*Edenton, N. C.*

